# A meta-analytic cognitive framework of nudge and sludge

**DOI:** 10.1098/rsos.230053

**Published:** 2023-11-29

**Authors:** Yu Luo, Andrew Li, Dilip Soman, Jiaying Zhao

**Affiliations:** ^1^ Department of Psychology, University of British Columbia, Vancouver, British Columbia, Canada; ^2^ Rotman School of Management, University of Toronto, Toronto, Canada; ^3^ Institute for Resources, Environment and Sustainability, University of British Columbia, Vancouver, British Columbia, Canada

**Keywords:** behavioural intervention, meta-analysis, cognition, behaviour change, policy

## Abstract

Public and private institutions have gained traction in developing interventions to alter people's behaviours in predictable ways without limiting the freedom of choice or significantly changing the incentive structure. A nudge is designed to facilitate actions by minimizing friction, while a sludge is an intervention that inhibits actions by increasing friction, but the underlying cognitive mechanisms behind these interventions remain largely unknown. Here, we develop a novel cognitive framework by organizing these interventions along six cognitive processes: attention, perception, memory, effort, intrinsic motivation and extrinsic motivation. In addition, we conduct a meta-analysis of field experiments (i.e. randomized controlled trials) that contained real behavioural measures (*n* = 184 papers, *k* = 184 observations, *N* = 2 245 373 participants) from 2008 to 2021 to examine the effect size of these interventions targeting each cognitive process. Our findings demonstrate that interventions changing effort are more effective than interventions changing intrinsic motivation, and nudge and sludge interventions had similar effect sizes. However, these results need to be interpreted with caution due to a potential publication bias. This new meta-analytic framework provides cognitive principles for organizing nudge and sludge with corresponding behavioural impacts. The insights gained from this framework help inform the design and development of future interventions based on cognitive insights.

## 1. Introduction

Behaviour change approaches have been extensively explored and tested in both public and private sectors to guide human choices. In the last few decades, one particular approach called nudge has gained significant traction, defined as a change in the choice architecture (i.e. the context in which choices are presented to people) that alters people's behaviours without limiting the freedom to choose or significantly changing the incentive structure [1]. Since the introduction of nudge, choice architects from public and private institutions have been developing and testing a variety of nudges to influence human decision making. More recently, several scholars have introduced a closely related concept, sludge, where the choice context impedes behaviour by increasing decision friction [2–4].

Although researchers have attempted to concretely define nudge and sludge, these terms remain equivocal. In this paper, we define *nudge* as an intervention that facilitates actions by reducing friction, while *sludge* is defined as an intervention that impedes actions by increasing friction. These definitions are based from the theorized forces that drive human behaviour from the force-field analysis developed by Kurt Lewin [5], which suggests that human behaviour is facilitated by driving forces that help people make progress toward a goal, but are hindered by restraining forces that prevent people from taking action. In addition to friction, another important dimension of nudge and sludge is that interventions facilitating or impeding actions can benefit or harm people [2,3]. For example, automatically enrolling employees into a pension plan helps them by facilitating their action of saving for retirement (i.e. a beneficial nudge). Presenting an ‘Are you sure?' warning message helps people by impeding their action of making an impulse purchase (i.e. a beneficial sludge). On the other hand, automatically subscribing to a magazine harms people by facilitating their action of paying for unnecessary fees (i.e. a harmful nudge), and filling out complex tax forms harms people by deterring them from getting a tax return (i.e. a harmful sludge). We used the term ‘beneficial sludge' rather than decision points or cooling off periods mentioned in Soman *et al*. [2] for three reasons. First, it is more parsimonious to classify interventions along two dimensions (beneficial versus harmful for consumers × reduce friction versus increase friction), than with four separate concepts. Second, since most behavioural scientists and policymakers are familiar with the terms nudge and sludge, introducing the idea that a nudge can be harmful, and a sludge can be beneficial can broaden the scope of nudge and sludge, without learning new terms like dark patterns and decision points. Finally, decision points and cooling off periods are very specific interventions designed to increase friction to benefit consumers. In reality, a beneficial sludge can be much broader than that.

Since most literature on nudge and sludge is geared towards solving real-world problems, past studies tend to prioritize developing and testing different interventions to show impact over understanding why an intervention works or fails [6], specifically the underlying cognitive mechanisms of an intervention that lead to its success or failure. Understanding the cognitive mechanism of a given intervention is crucial for ensuring the efficacy of the intervention. During the development of an intervention, a typical approach is to first identify a target behaviour to change, then diagnose the behaviour by examining its underlying barriers and motivators (i.e. restraining and driving forces), followed by finding an appropriate change in the choice context that can either remove the barrier or increase the motivation to change the target behaviour [7]. Diagnosing the barriers and motivators of the behaviour often relies on cognitive insights and processes [8]. For example, barriers to recycling include a lack of attention (e.g. not noticing the recycling bins), too much effort (e.g. recycling is inconvenient), and a failure of memory (e.g. forgetting to recycle). Motivators of recycling include intrinsic motivation (e.g. a desire to be environmentally friendly) and extrinsic motivation (e.g. getting $0.10 back per recycled bottle). Despite the importance of cognitive insights, no study to date has systematically examined the cognitive mechanisms of nudge and sludge. Moreover, there are no systematic comparisons of the effectiveness of nudge and sludge under a common framework; therefore it is unknown whether nudge or sludge is more effective in changing behaviour.

To address these knowledge gaps, we propose a new cognitive framework that categorizes interventions that facilitate or impede actions based on six key cognitive processes. In addition, we conduct a meta-analysis to examine the effect size of the interventions targeting each cognitive process, with the goal of identifying which intervention is the most effective.

## 2. A cognitive framework

Our cognitive framework is organized along three dimensions: the first dimension is the type of intervention (facilitating versus impeding actions); the second is whether the intervention benefits or harms people; and the third dimension is the cognitive processes involved in eliciting the desired behaviour change intended by the intervention (see table 1 for definitions of cognitive processes and a list of examples of interventions and additional explanations for each intervention in table S1 in §A of electronic supplementary material).

**Table 1.** Definitions of the six cognitive processes and examples of interventions that benefit or harm people by facilitating or impeding actions.

cognitive process (definition)	benefit people		harm people	
	facilitate actions	impede actions	facilitate actions	impede actions
**attention** (using stimulus features to increase or decrease the salience of an option)	• abrupt onset	• ‘are you sure’ alert	• bright colours (e.g. brand logo)	• concealment
	• cueing	• salient warning	• sensory cues in casinos (e.g. flashing lights)	• distracting notification
	• highlighting	• increased font size of calories label		• reduced font size
	• visibility			
**perception** (framing the content of information to influence the conscious interpretation of the information)	• appearance	• loss framing	• bundle pricing	• decoy option
	• assortment size	• smaller portions		• price partitioning (e.g. taxes, shipping fees)
	• availability			
	• feedback			
	• gain framing			
	• accessible graphics			
	• information			
**memory** (using encoding cues or retrieval cues to alter subsequent decisions)	• anchoring (e.g. suggested donation amount)	• reminder (e.g. reducing water consumption)	• anchoring (e.g. maximum deposit amount)	• absence of reminder at the end of trial periods
	• reminder (e.g. promoting college enrollment)		• repetitive advertising	
	• priming		• subliminal advertising	
	• visual prompt			
**effort** (changing cognitive or physical ease associated with an option)	• accessibility	• active choice	• accessibility to unhealthy food	• complex cancellation procedures
	• convenience	• inconvenience	• convenience (e.g. tabletop ATMs in casinos)	• mail-in rebates
	• default		• default (e.g. overdraft protection)	• non-transparent privacy settings
	• simplification			
**intrinsic motivation** (influencing inherent interests toward an option in the absence of external factors)	• commitment making	• self-control tools	• junk food advertising that triggers internal drives	• vaping norms for smokers who want to quit
	• goal setting	• social norm (e.g. reducing electricity consumption)	• vaping norms for non-smokers	
	• implementation intention			
	• motivational intervention			
	• social norm (e.g. promoting donation)			
**extrinsic motivation** (imposing small external rewards or punishments to alter decisions)	• financial incentives	• conditional incentives	• micro-incentives to gamble	• membership fees
	• non-financial incentives (e.g. smiley stamps)	• small fees for no-shows		

The six cognitive processes are motivated by the pioneering work of Maule [9] who proposed that cognitive psychology serves as a foundation for decision making research. In particular, decision making is determined by different stages of information processing, from encoding sensory inputs which are heavily influenced by attention, to consolidating and storing inputs in memory. Moreover, Maule [9] suggested that motivation is an important factor in decision making, but cognitive psychology tends to neglect it. Inspired by these insights, we will discuss how each cognitive process is targeted in the interventions in the next section. The six cognitive processes were not mutually exclusive as multiple cognitive processes could be involved in a specific intervention. Moreover, this framework was intended to be a starting point for researchers and practitioners to build on in future studies.

An attention intervention in the current framework is defined as *an intervention that uses stimulus features to increase or decrease the salience of an option*. This is supported by the theories proposing that attention is controlled by top-down and bottom-up factors [10–12]. However, bottom-up attention is relatively easier to target by using salient stimuli to exogenously draw attention, such as colour [13], motion [14], size [15] and abrupt onset [16]. For example, drawing attention to the licence renewal message helps people by facilitating their renewal actions [17]. Colour can also help people by holding them back from potential risks, such as a red warning sign before opening a phishing website [18]. A harmful intervention that facilitates actions is red and yellow colours frequently used as part of fast-food brand logos to draw people's attention, which may increase the temptation for junk food [19]. Another harmful intervention is displaying resort fee disclosures in a small font to deter people from noticing the fees. We recognize that the same intervention may carry different meanings under different study contexts. In this paper, we only intend to discuss some examples of interventions for each cognitive process rather than providing a comprehensive list of interventions in all possible contexts.

A perception intervention is defined as *an intervention that frames the content of information to influence the conscious interpretation of the information*. Perception is known as the organization, identification and interpretation of sensory inputs to create a mental representation of external information [20]. This is a necessarily narrow definition of perception based on existing nudge studies that aimed to shift perception by reconstructing information with a specific frame to influence subsequent behaviours. A popular perception intervention is framing the benefits or costs of an action, such as framing the beneficial outcomes of climate change mitigation to facilitate pro-environmental actions [21] or displaying adverse consequences of smoking on cigarette packages to impede smoking [22,23]. Framing can also be used for harm. In a field experiment, Ganzach & Karsahi [24] found that conveying the losses of not using a credit card to cardholders doubled the percentage of cardholders starting to use their credit, and more than doubled the expenditures of these cardholders. This framing intervention is harmful as it facilitates overspending. A harmful intervention that impedes actions is to add a decoy option inferior to a target option, preventing people from choosing the option best suited to their needs [25].

A memory intervention is defined as *an intervention that uses encoding cues or retrieval cues to alter behaviours*. This definition is supported by the multi-store model of memory which explains how external information is transferred and stored into long-term memory [26]. Priming and anchoring interventions can influence the encoding process to enhance the registration of new information; however, many of these studies have not been well replicated (e.g. [27,28]). Choice architects often use retrieval cues, such as reminders or visual prompts, to encourage or discourage subsequent actions. For example, visual prompts can encourage people to turn off lights in unoccupied washrooms [29,30]. Visual prompts also deter people from making undesirable decisions, such as signs that ask people to rethink their reason for smoking [31]. Moreover, a potentially harmful memory intervention is the absence of reminders before the end of a free trial which impedes the action of cancelling by preventing people from remembering to perform an important action.

An effort intervention is defined as *an intervention that modifies the cognitive or physical ease associated with an option*. The definition of effort is derived from ‘the law of less work' suggesting that people tend to choose an option that requires minimum cognitive or physical effort [32,33], and people prefer to maintain the status quo instead of switching to an alternative choice [34–37]. For example, a default intervention facilitates employees' savings by automatically enrolling them in a retirement savings plan [38]. In contrast to default, active choice requests people to explicitly accept or decline an option. Hedlin & Sunstein [39] showed that when participants were told that green energy was more expensive, active choosing was associated with a higher enrollment rate than the default. Another effort intervention is convenience, but it can be used against people's interests as a harmful intervention. For instance, modern gambling machines with touchscreen buttons require less physical effort during long gambling sessions, compared to traditional machines with a lever [40]. Complex redemption rules for mail-in rebates are an example of effort intervention that harms people by deterring redemption.

An intrinsic motivation intervention is defined as *an intervention that influences the inherent interests toward an option in the absence of external factors*. This definition is derived from the self-determination theory [41]. Interventions that facilitate actions aim to increase people's inherent interest in engaging in new behaviours, and those that impede actions undermine people's inherent interests to deter them from undesirable behaviours. Importantly, we conceptualize all internalized forms of extrinsic motivation as intrinsic motivation to more clearly distinguish intrinsic from extrinsic motivation [42]. Goal setting has been used as an intervention that turns intrinsic motivation into actions by identifying clear goals. Social norm messaging has been used as a popular intervention to facilitate actions by increasing the intrinsic motivations to improve past behaviours based on information on how others are doing.

In contrast to intrinsic motivation, an extrinsic motivation intervention is *an intervention that imposes small external rewards or punishments to alter behaviours*. Since nudge is thought to not significantly change the incentive structure [1], interventions providing small financial and non-financial rewards or punishments are labelled as extrinsic motivation interventions in the current framework. This said, what counts as ‘small' is still debatable in the literature, and there is no clear threshold for significantly changing the incentive structure in the original conceptualization of nudge [1]. In the current framework, extrinsic motivation interventions that facilitate actions provide small incentives to promote desirable actions, while interventions that impede actions impose small punishments to deter undesirable actions. One example is to provide a small financial reward to facilitate actions or impose financial penalties to deter actions. Financial incentives can also be used as a harmful intervention that facilitates actions to induce impulsive behaviours, such as online gambling platforms that offer sign-up incentives to attract gamblers. Moreover, charging costs associated with an option can be a harmful intervention that impedes actions, such as retailers charging a membership fee to prevent customer attrition.

Since our framework aimed to target a broader audience from different fields, we did not limit our examples discussed above to a single context. However, a recent study designed an intervention for each cognitive process to reduce single-use produce bags and tested the efficacy of the interventions with the same dependent measure. The study showed that participants in all intervention conditions used fewer produce bags than those in the control condition, but the efficacy in reducing produce bags varied across interventions and cognitive processes [43]. This study can be used as an example of how to design different interventions based on different cognitive processes in the same context.

## 3. Meta-analysis

We have thus far outlined the cognitive framework and provided examples of interventions targeting the six cognitive processes. As a critical empirical evaluation of the framework, we will examine the effect size of these interventions by conducting a meta-analysis. There are only a few existing meta-analyses on nudge. One meta-analysis [44] on healthy eating showed that interventions targeting motor responses (e.g. increasing ease of accessing healthy foods) had a larger effect size than interventions that influence knowledge (e.g. nutrition labelling) or feeling (e.g. attractive graphics). Two additional meta-analyses examined interventions across multiple domains, such as energy, environment, finances, health, and policy-making, and found that default had the largest effect size among other interventions [45], and that interventions that used automaticity had a larger effect size than those that did not [45,46]. A recent meta-analysis also found that interventions on choice alternatives (e.g. default) outperformed those that reinforce behavioural intentions [47,48].

The existing meta-analyses had several limitations. First, the meta-analyses included mixed interventions that combined multiple interventions in a single condition, which makes it impossible to identify the impact of a single intervention. Second, the meta-analyses included a mixture of self-reported surveys, laboratory studies, and field experiments, which makes it difficult to tease out the causal effect of interventions on real behaviours [49,50]. Third, the meta-analyses mixed the results of quasi-experiments with randomized controlled trials, which makes it difficult to cleanly identify the causal factor. Finally, the meta-analyses did not separate nudge from sludge, therefore could not identify whether interventions that facilitate or impede actions had different impacts on behaviour. To address these limitations, we conduct a meta-analysis with only field experiments (i.e. randomized controlled trials) with actual behavioural measures instead of self-reports to examine the effectiveness of interventions targeting each of the six cognitive processes outlined in the cognitive framework.

### 3.1. Methods

All of the data and code of the meta-analysis are available here: https://doi.org/10.7910/DVN/N9EJNR [51]. To create the dataset, we conducted a literature search in seven databases across multiple disciplines: Web of Science, PubMed, PsychInfo, Business Source Ultimate, PsychExtra, Google Scholar and Proquest. The last two databases were used to include grey literature, such as non-academic articles, business reports and unpublished dissertations. The first search term was ‘nudge', ‘nudging', ‘sludge’ or ‘sludging', and the second joint search term was ‘randomized controlled trial'. Given the large number of search results on Google Scholar, the second term was changed to ‘field experiment' to limit the number of articles. Moreover, disciplines such as physics and meteorology that use the terms nudge and sludge based on other definitions were excluded from the search. The publication year was restricted from 2008 to 2021 as the term nudge was popularized after the publication of the book *Nudge* in 2008 [1].

After removing duplicates from the initial search, we analysed the titles and abstracts to exclude review articles or studies reporting qualitative data. In the full-text assessment stage, articles were selected based on four criteria: field experiments, single interventions, randomized controlled trials and actual behavioural measures. Articles that tested multiple interventions in separate conditions were included as distinct observations in the initial dataset. However, for these studies (*n* = 32), we randomly chose one of the interventions using a randomization tool in R and removed the other interventions in our analysis (*k* = 41 removed) to avoid overweighting studies from a specific paper. For articles that examined the same intervention on different samples, the pooled effect was calculated across samples. Articles that used mixed interventions (e.g. reminders with social norm messaging) in one condition were excluded from the analysis. Actual behavioural measures were defined as objective measures of behaviours (e.g. actual per cent change in energy consumption) rather than self-reported behaviours.

**Figure 1.**
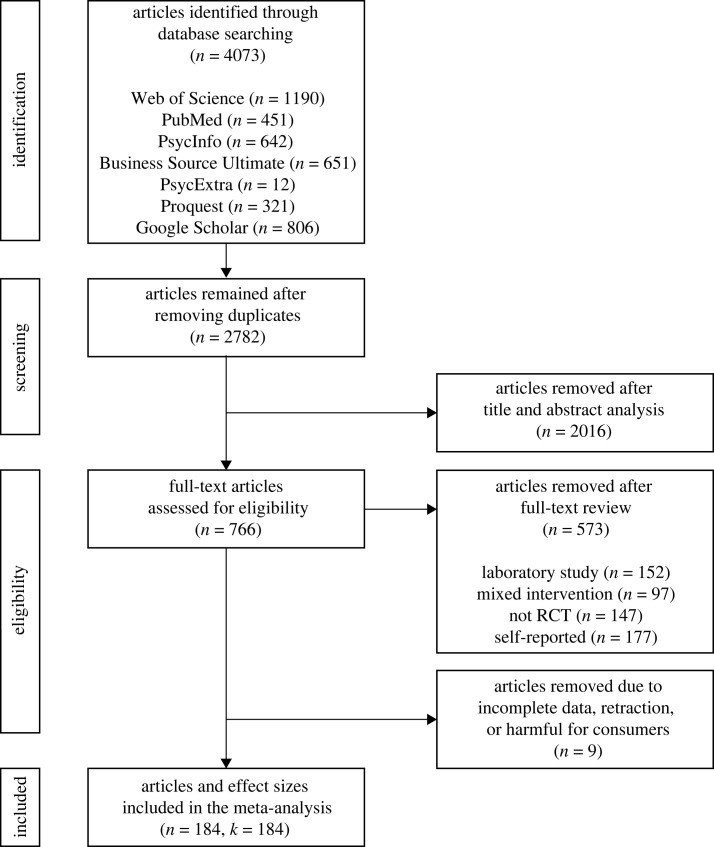
A PRISMA flow diagram showing the four stages of article selection with the number of articles in each stage.

In searching for the articles, we only found three published articles on nudge and one on sludge that harmed people. Specifically, among the three articles on harmful nudge, one used a memory intervention (i.e. priming, *d* = 0.17; and financial incentives, *d* = 0.20 [52]) and two intrinsic motivation interventions (i.e. both social norms messaging, *d* = 0.54 and *d* = 0.91 [53,54]), and the article on harmful sludge used an attention intervention (i.e. visibility, *d* = 0.02 [55]). We only found a total of five effect sizes from four articles possibly due to the ethical concerns with conducting studies with harmful interventions, or to the unavailability of data from authors. Thus, a systematic comparison between interventions that benefit and harm people was not feasible. We therefore excluded the five effect sizes from four articles that involved harmful interventions from the meta-analysis. In other words, the current meta-analysis only contained interventions that benefited people. In total, *n* = 184 articles met all criteria, and *k* = 184 observations and *N* = 2 245 373 participants were included in the meta-analysis (see details of the selected articles in §B of electronic supplementary material). Figure 1 presents the PRISMA flow diagram showing the four stages of article selection with the number of articles at each stage.

**Figure 2.**
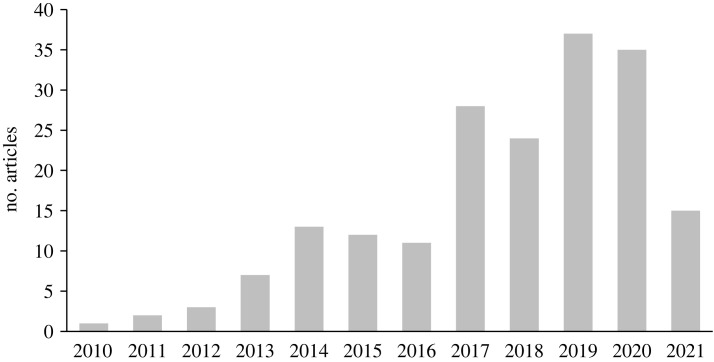
The number of selected articles per year in the meta-analysis.

Since we did not limit our search to a specific discipline, studies from the education, environment, finance, health and policy-making sectors were included in the meta-analysis. By analysing the number of articles published per year among the articles included in the meta-analysis, more articles were published from 2017 to 2021, showing an increased interest in examining the effect of nudge and sludge on actual behaviour change in field experiments (figure 2). A decrease in the number of field experiments published in 2021 could be due to the lockdowns in the COVID-19 pandemic.

The 184 observations were further categorized into one of the six cognitive processes based on the definitions discussed in the cognitive framework (table 1). Specifically, the definition of each cognitive process from the first column of table 1 was provided to two coders for them to learn the concept of each cognitive process in the current framework. Then, the two coders independently categorized each intervention into one of the six cognitive processes. Each intervention was classified as nudge or sludge, depending on whether the intervention facilitates or impedes actions. There was a 95% agreement between the two coders. For the 5% of disagreement, the coders discussed their reasons for coding the interventions, and eventually reached an agreement after the discussion. In addition, we categorized different nudge and sludge techniques into intervention categories based on the short guide by Sunstein [56]. For techniques that did not fit into the 10 important nudges, we collected the label of the new technique given by the author and combined those sharing similar features into the same intervention category.

When an article reported multiple outcome variables, we collected the values of the primary outcome variable stated by the authors. If the primary outcome variable was not explicitly declared, we chose the first outcome variable analysed and discussed by the authors. Since different studies used different outcome measures, we converted the various effect sizes to a standardized measure following the guidelines provided by Harrer *et al.* [57]. Specifically, we converted the mean difference between the treatment condition and the control condition to Cohen's *d* by dividing the pooled standard deviation for continuous variables. If a study did not provide sufficient information on the means, standard deviations and sample sizes, the raw data were requested and obtained, and manually analysed to obtain the relevant statistics. Studies that failed to report the complete set of statistics and did not provide the raw data were excluded. When studies used dichotomous variables, the odds ratio was computed and then converted to Cohen's *d*. Several studies used dummy coding for the dichotomous variable and reported the relative difference between the treatment condition and the control condition, and the relative difference was converted to Cohen's *d*. Several studies reported a raw comparison between the treatment and control condition, and additional comparisons after controlling for covariates. To minimize biases in the results, only comparisons without controlling for covariates were included in the meta-analysis. Moreover, some observed reductions in undesirable behaviours (e.g. reduced water consumption) were coded as positive although the original effect size was negative.

### 3.2. Results and discussion

After pooling the effect sizes using the random-effects model, the overall effect size was 0.28 (Cohen's *d*) from the meta-analysis. The overall heterogeneity was estimated to be *I*^2^ = 99.7% which suggests substantial variability in the effect sizes of the interventions. We further conducted a subgroup analysis by introducing the type of intervention (nudge versus sludge) in our model. Nudge (interventions that reduce friction, *k* = 144) had an average effect size of 0.29 (*I*^2^ = 98.2%), and sludge (interventions that increase friction, *k* = 40) had an average effect size of 0.26 (*I*^2^ = 99.9%). Across the six cognitive processes (table 2), effort interventions had the largest effect size (*d* = 0.52, *I*^2^ = 96.8%), followed by extrinsic motivation interventions (*d* = 0.31, *I*^2^ = 90.2%), perception interventions (*d* = 0.27, *I*^2^ = 98.4%), attention interventions (*d* = 0.20, *I*^2^ = 96.2%), memory interventions (*d* = 0.20, *I*^2^ = 92.5%) and intrinsic motivation interventions (*d* = 0.17, *I*^2^ = 99.9%).

**Table 2.** Effect size (Cohen's *d*) of interventions that reduce friction (i.e. nudge) or increase friction (i.e. sludge) by cognitive processes.

cognitive process	type	*k*	*d* [95% CI]	combined *d* [95% CI]
attention	nudge	10	0.18 [0, 0.36]	0.20 [0.04, 0.35]
	sludge	3	0.29 [−0.66, 1.24]	
perception	nudge	32	0.30 [0.15, 0.45]	0.27 [0.15, 0.38]
	sludge	10	0.19 [0.04, 0.34]	
memory	nudge	34	0.19 [0.12, 0.27]	0.19 [0.13, 0.26]
	sludge	2	0.22 [0.05, 0.38]	
effort	nudge	26	0.57 [0.32, 0.82]	0.52 [0.32, 0.72]
	sludge	9	0.36 [0.03, 0.69]	
intrinsic motivation	nudge	27	0.12 [0.04, 0.2]	0.17 [0.05, 0.29]
	sludge	12	0.24 [−0.13, 0.61]	
extrinsic motivation	nudge	15	0.32 [0.12, 0.52]	0.31 [0.14, 0.47]
	sludge	4	0.27 [−0.26, 0.8]	
overall	nudge	144	0.29 [0.22, 0.35]	0.28 [0.22, 0.34]
	sludge	40	0.26 [0.12, 0.39]	

**Figure 3.**
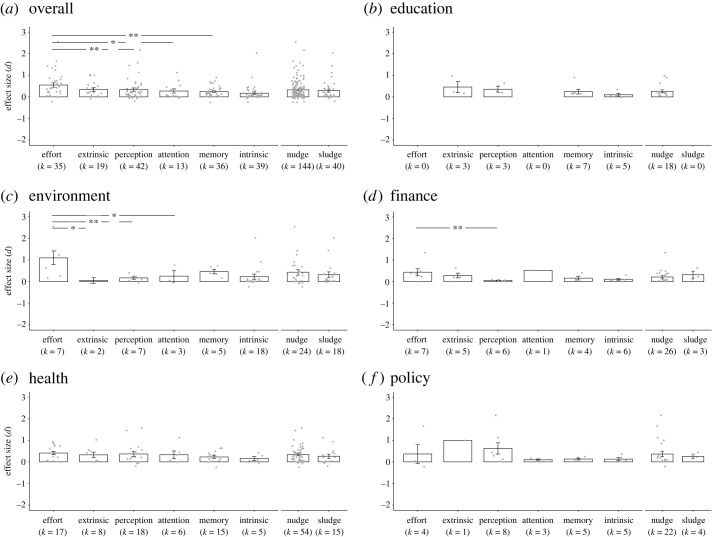
Comparisons of effect sizes across the six cognitive processes overall (*a*) and in each domain (*b–f*) (**p < 0.01, *p < 0.05, error bars mean 1 ± s.e.). *k =* 0 means that there was no observation in that category.

To examine differences in effect size across the six cognitive processes, a univariate meta-regression was conducted (figure 3*a*). This analysis showed that the effect sizes differed significantly across the cognitive processes (*F*_5,178_ = 3.13, *p* = 0.01). *Post hoc* Tukey's HSD tests were conducted that showed that effort interventions had significantly larger effect sizes than memory interventions (*p* = 0.003), perception interventions (*p* = 0.008), and attention interventions (*p* = 0.02). We found no significant difference between nudge and sludge (*F*_1,182_ = 0.17, *p* = 0.68; figure 3*a*).

Moreover, we examined the effect sizes across the cognitive processes by domain (figure 3*b–f)*. Interventions targeting environmental decisions (*d* = 0.37, *I*^2^ = 99.9%) had the largest effect size, followed by policy decisions (*d* = 0.31, *I*^2^ = 99.4%), healthcare decisions (*d* = 0.26, *I*^2^ = 81.4%), educational decisions (*d* = 0.21, *I*^2^ = 88.5%) and financial decisions (*d* = 0.21, *I*^2^ = 98.8%). In each domain, we also conducted a univariate meta-regression to compare effect sizes across the cognitive processes. In the environment domain (figure 3*c*), there was a significant difference between cognitive processes (*F*_5,36_ = 2.80, *p* = 0.03), and *post hoc* Tukey's HSD tests showed that effort interventions had significantly larger effect sizes than perception interventions (*p* = 0.003), extrinsic motivation interventions (*p =* 0.02), and attention interventions (*p* = 0.02). In the finance domain (figure 3*d*), there was a significant difference between cognitive processes (*F*_5,23_ = 2.85, *p* = 0.04), and *post hoc* Tukey's HSD tests showed that effort interventions had significantly larger effect sizes than perception interventions (*p* = 0.003). However, there was no significant difference between the cognitive process in the education (*F*_3,14_ = 0.89, *p* = 0.47), health (*F*_5,63_ = 0.59, *p* = 0.71) and policy domains (*F*_5,20_ = 1.07, *p* = 0.41) (figure 3*b*,*e*,*f*, respectively). We further examined the effect sizes between nudge and sludge and found no significant difference in the environment (*F*_1,40_ = 0.41, *p* = 0.53), finance (*F*_1,27_ = 0.55, *p* = 0.47), health (*F*_1,67_ = 0.89, *p* = 0.35) and policy domains (*F*_1,24_ = 0.05, *p* = 0.83).

To examine which specific intervention had the largest effect size, we conducted a univariate meta-regression on the interventions that had at least two observations in the meta-analysis. This is because a minimum of two data points per intervention was required to conduct the meta-regression (table 3). There was a significant difference between these interventions (*F*_26,152_ = 1.85, *p* = 0.01) (see *post hoc* test results in §C of electronic supplementary material). Numerically speaking, convenience (*d* = 1.18), changing the appearance of an option (*d* = 0.81), rewarding with non-financial incentives (e.g. stamps with smiley faces, *d* = 0.39) making a commitment (*d* = 0.36), using priming (*d* = 0.31), and highlighting information (*d* = 0.27) had the largest effect sizes in interventions targeting effort, perception, extrinsic motivation, intrinsic motivation, memory and attention, respectively (table 3). This said, these results need to be interpreted with caution due to the small number of studies in each category.

**Table 3.** Effect sizes by interventions.

intervention	cognitive process	*k* (>1)	*d* [95% CI]
convenience	effort	3	1.18 [−0.38, 2.74]
appearance	perception	2	0.81 [−5.84, 7.47]
inconvenience	effort	5	0.67 [0.14, 1.19]
default	effort	14	0.58 [0.17, 1.00]
accessibility	effort	7	0.43 [0.16, 0.69]
informational feedback	perception	5	0.39 [0.25, 0.54]
non-financial incentives	extrinsic	5	0.39 [−0.03, 0.82]
conditional incentives	extrinsic	3	0.37 [−0.45, 1.2]
availability	perception	5	0.36 [−0.36, 1.08]
commitment making	intrinsic	5	0.36 [−0.04, 0.76]
informational messaging	perception	11	0.35 [0.11, 0.60]
priming	memory	6	0.31 [−0.06, 0.68]
gain framing	perception	8	0.3 [−0.16, 0.76]
financial incentives	extrinsic	10	0.28 [0.01, 0.55]
highlighting	attention	3	0.27 [−0.6, 1.14]
graphic	perception	2	0.26 [−3.63, 4.15]
anchoring	memory	2	0.26 [−3.03, 3.54]
goal setting	intrinsic	2	0.2 [−1.52, 1.93]
reminder	memory	28	0.17 [0.11, 0.23]
social norm	intrinsic	24	0.16 [−0.02, 0.35]
visibility	attention	7	0.16 [−0.06, 0.37]
motivational intervention	intrinsic	3	0.1 [−0.09, 0.29]
active choice	effort	4	0.08 [−0.07, 0.24]
loss framing	perception	5	0.04 [−0.02, 0.10]
implementation intention	intrinsic	5	0.03 [−0.02, 0.09]
simplification	effort	2	0.03 [−0.12, 0.18]
assortment size	perception	3	<0.01 [−0.5, 0.51]

**Figure 4.**
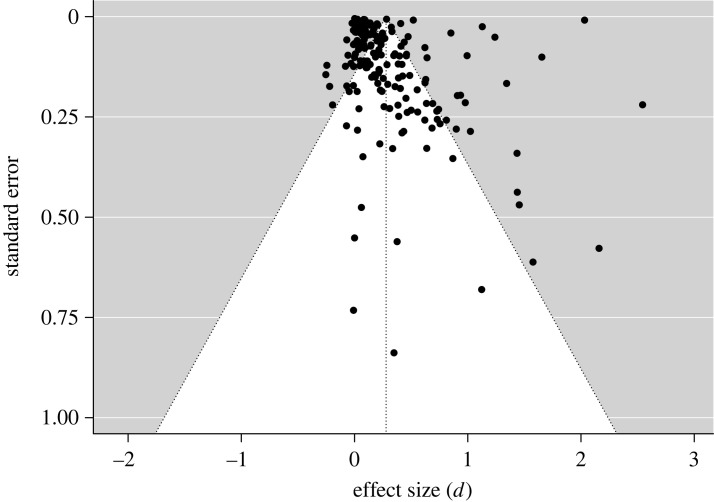
The funnel plot appears to be asymmetrical.

To examine any publication bias in the meta-analysis, a funnel plot was created. Egger's test showed that there was a significant asymmetry in the funnel plot (*z* = 5.37, *p* < 0.001; figure 4), suggesting that a publication bias in reporting successful interventions over unsuccessful interventions may exist. The publication bias found here was consistent with the recent meta-analysis on the effectiveness of nudge that also showed a publication bias [47,48].

Given the recent discussions on the weak effect of nudge after adjusting for publication bias [58–60], we used two publication bias adjustment methods in our meta-analysis: selection models and robust Bayesian meta-analysis. The selection models method reduced the overall effect size (*d*) from 0.28 to 0.33 which remained as a small to medium effect size. The robust Bayesian meta-analysis method detected a null overall effect of *d* = −0.005. Thus, the adjusted overall effect of the interventions varied based on the specific selection of adjustment methods (also see the adjusted effect sizes split by cognitive process in §D of electronic supplementary material).

## 4. General discussion

The current paper first proposed a new cognitive framework to categorize behavioural interventions that facilitate actions (nudge) or impede actions (sludge), benefit or harm people, by six cognitive processes. It provides an organizing framework to categorize interventions based on cognitive insights. This framework is valuable for at least four reasons. First, it helps facilitate the diagnosis of the underlying barriers and motivators of a given behaviour by illuminating the six cognitive dimensions relevant to decision making. Second, it helps identify an appropriate intervention targeting specific cognitive processes to remove the barriers or enhance the motivation of a given behaviour. For example, if the choice architect identifies that a lack of attention is a behavioural barrier, they can use the interventions in the attention category to change behaviour. Third, the framework offers a number of interventions to increase or decrease decision friction per cognitive process, allowing choice architects to choose an intervention from the existing set or tailor an intervention to the choice context. Finally, the framework highlights not only the potential benefit of interventions that help people take the desired action, but also the potential harmful interventions that are detrimental to people's welfare and should be avoided in practice.

The current framework is more comprehensive compared to past frameworks. For example, it overlaps with the nine categories in the MINDSPACE framework [61]. However, effort, memory and perception interventions in the current framework are more inclusive than default, priming, affect and messenger interventions in MINDSPACE. For example, effort interventions include not only default but also interventions manipulating the degree of convenience. Memory interventions contain reminders in addition to priming, and perception interventions extend to informational and real-time feedback. Likewise, the current framework includes similar interventions to the EAST framework [62], but offers cognitive insights on Easy (effort), Attractive (attention), Social (intrinsic motivation) and Timely (memory). Additionally, our framework shares several similarities with the recent TIPPME framework that focuses on how interventions can alter the properties (attention, perception) and the placements (effort) of the choices to change behaviours [63], but also includes other types of interventions. Therefore, the framework provides systematic insights on how interventions facilitate or impede actions through which cognitive processes.

The current paper also examined the effect size of the interventions targeting each cognitive process in a meta-analysis containing only field experiments with real behavioural measures. The meta-analysis showed that the interventions that targeted effort (e.g. convenience, default) had the largest effect size compared to the other interventions. This finding was supported by previous meta-analyses that demonstrated default and automaticity interventions were the most effective [45,46]. Interventions targeting intrinsic motivation (e.g. goal setting, implementation intention, social norms) had the smallest effect size in comparison. The effect size of intrinsic motivation interventions found in the current meta-analysis was comparable to the findings of a recent meta-analysis of randomized controlled trials on pro-environmental behaviours [64].

The results of the meta-analysis help rank the efficacy of interventions in previous frameworks. For example, the authors of MINDSPACE claimed that the ordering of the nine categories in the acronym was not meaningful. According to the current meta-analysis, the nine categories can be ordered based on the effectiveness of the interventions. Specifically, choice architects should consider default (effort) as their first intervention, then incentives (extrinsic motivation), messengers and affect (perception), salience (attention), priming (memory), and lastly ego, commitment and norms (intrinsic motivation) interventions.

Since the meta-analysis included interventions from multiple domains, the average effect size of interventions varied across domains, which is consistent with Mertens *et al*.'s meta-analysis [47,48]. We found that effort interventions had the largest effect size in the environment, health, and finance domains, but extrinsic motivation interventions had the largest effect size in the education and policy domains. This said, the current findings by domain need to be interpreted with caution given the small sample size in each domain.

The current meta-analysis was the first to compare the impact of nudge and sludge and found that the effect sizes of nudge and sludge were not significantly different. This suggests that interventions that facilitate actions and those that impede actions had similar efficacy in promoting behaviour change. However, the sample size of interventions that impede actions was considerably smaller (*k* = 40) than that of interventions that facilitate actions (*k* = 144), suggesting that the effect size of interventions that impede actions should be interpreted with caution. This also calls for the need for more research to examine the impact of interventions that impede actions.

Across the subgroup analyses by the type of intervention, cognitive process and domain, we found considerable heterogeneity in the effect size of interventions, which is consistent with the meta-analysis by Mertens *et al*. [47,48]. It is critical for behavioural scientists to investigate the variability in the effect of interventions by identifying other moderators that could explain the failure and success of a given intervention in a specific context [47,48,65,66]. This will help practitioners design the optimal interventions tailored to different audiences with diverse views, values, and information needs.

These results are promising, but we found a potential publication bias in reporting successful interventions over unsuccessful ones. This finding was consistent with the recent meta-analysis [47,48]. After adjusting for publication bias using different methods, the adjusted overall effect of the interventions varied based on the specific selection of adjustment methods. Thus, practitioners should be aware of the impact of adjustment methods on the effect sizes and the generalizability of these estimates reported in the current meta-analysis.

This new meta-analytic cognitive framework has several theoretical, empirical, and practical contributions. First, it provides cognitive insights on nudge and sludge by explaining which cognitive process is involved in a given intervention. Second, the framework allows comparisons of impact between interventions that target different cognitive processes. For example, reducing effort by making an option more convenient or drawing attention by highlighting the option was more impactful in achieving behaviour change than increasing intrinsic motivation by using social norm messaging. Third, the meta-analysis excluded self-reported data and laboratory studies, permitting comparisons using only behavioural measures in field experiments, which is important to demonstrate real-world impact. Fourth, since only randomized controlled trials were included in the analysis, the effect of the interventions demonstrates a causal impact of the interventions on real behaviour change. Finally, the framework could offer a ranking of interventions based on cognitive processes and the associated behavioural impact, which can guide the development of future interventions. However, this ranking needs to be interpreted with caution given the publication bias and selection bias in the type of interventions being tested in the behavioural science literature.

Although the theoretical, empirical and practical contributions of the current review are prominent, the current framework has some limitations. First, we defined each cognitive process using one of the many definitions in cognitive science. This choice was strategic because we wanted to avoid having vague or overly broad definitions that would make classifying the interventions intractable. As a result, we could not include other theoretical definitions of each process in our framework. Second, the categorization of the interventions based on the cognitive processes and the type of intervention (facilitating versus impeding actions) was subjective. Future studies can seek further support for the categorization with empirical data, for example, by inviting other researchers who are familiar with cognitive concepts to classify the interventions based on the definitions discussed in the current review. The consensus among these experts will reduce the subjectivity of this cognitive framework. Third, some interventions may contain more than one cognitive process (e.g. a visual reminder may draw attention and reduce forgetting at the same time). Future studies can conduct a detailed analysis of how different cognitive processes are involved in a specific intervention. Fourth, in the meta-analysis, 91% of the selected studies were conducted in developed countries which limits the generalizability of the effects of nudge and sludge to developing countries. Lastly, all studies included in the meta-analysis were based on information that was available online and passed the inclusion criteria. Thus, the effect size estimates reported here need to be interpreted with caution. We developed our meta-analytic framework to provide a starting point for practitioners to take into account the cognitive mechanisms underlying different interventions.

In conclusion, the current meta-analytic cognitive framework provides new insights on how nudge and sludge can be categorized along cognitive dimensions and it also demonstrates the effectiveness of the interventions targeting each cognitive process. This review paper can help inform the development of future behavioural interventions.

## Data Availability

The datasets generated and/or analysed during the current study are available in the Dataverse repository: https://doi.org/10.7910/DVN/N9EJNR [51]. Supplementary material is available online [67].
